# Predictive Factors for Cervical Intraepithelial Neoplasia in Women with Abnormal Cytology According to Human Papillomavirus Genotype: An Observational Study

**DOI:** 10.3390/ijms26199612

**Published:** 2025-10-01

**Authors:** Gonzalo Arturo Medina Bueno, Enrique Adolfo Jaramillo Saavedra, Natalia Torres Rendón, Damaris Diana Huareccallo Suni

**Affiliations:** 1 Department of Obstetrics and Gynecology, Faculty of Medicine, National University of San Agustin, Arequipa 04000, Peru; ejaramillo@unsa.edu.pe (E.A.J.S.); ntorresr@unsa.edu.pe (N.T.R.); dhuareccallo@unsa.edu.pe (D.D.H.S.); 2 Carlos Alberto Seguín Escobedo National Hospital of Essalud, Arequipa 04510, Peru

**Keywords:** human papillomavirus, HPV, cervical cytology, cervical intraepithelial neoplasia, CIN, genotype, colposcopy

## Abstract

Cervical cancer remains a leading cause of mortality among women, particularly in regions with limited resources. Persistent high-risk human papillomavirus (HR-HPV) infection is the main etiological factor for CIN and cervical cancer. This study aimed to evaluate the association between HPV genotypes, age, and cytological findings and the presence of CIN2–3 in women presenting with abnormal cervical cytology. This cross-sectional study included 189 women with abnormal cytology who attended a tertiary center in Peru. All participants underwent partial HPV genotyping using the Cobas 4800 assay, colposcopic evaluation, and colposcopically directed biopsies, which served as the diagnostic reference. Sociodemographic characteristics and reproductive histories were also collected. Multiple logistic regression was performed to assess the associations among specific HPV genotypes, age, cytological results, and CIN2–3 outcomes. Most participants were between 30 and 59 years of age (76.7%), and multiparity was common (77.8%). The most frequent cytological abnormalities were ASC-US (36.0%) and LSIL (28.0%), followed by HSIL (20.1%). HPV16 was detected in 24.3% of cases, HPV18 in 2.1%, and other HR-HPV types in 73.6%. HSIL cytology showed high concordance with histological CIN2–3 (>95%). Logistic regression demonstrated that age ≥ 30 years (aOR 4.50, 95% CI 1.90–10.65) and HPV16 infection (aOR 4.19, 95% CI 1.95–9.00) were the strongest independent predictors of high-grade disease. HPV18 was rare and not significantly associated, whereas other HR-HPV types showed an inverse association with CIN2–3. HPV16 and age ≥ 30 years were the most significant predictors of CIN2–3 in women with abnormal cytology, underscoring the dominant oncogenic role of HPV16. Integrating HPV genotyping, cytological findings, and age into risk-stratified algorithms could optimize cervical cancer prevention, ensuring timely detection of high-grade lesions while minimizing overtreatment in low-risk populations.

## 1. Introduction

Cervical cancer remains a significant public health concern worldwide, particularly in low- and middle-income countries, where it is one of the leading causes of cancer-related mortality among women [1]. The burden of this disease is especially high in regions such as Latin America, sub-Saharan Africa, and South Asia, where early detection rates and human papillomavirus (HPV) vaccination coverage are limited [2]. Cervical intraepithelial neoplasia (CIN) represents a spectrum of precancerous lesions that frequently precede invasive cervical carcinoma. Early detection and timely treatment of CIN are therefore key strategies in cervical cancer prevention.

HPV is a sexually transmitted virus that infects the skin and mucosal membranes of the anogenital tract. Persistent infection with high-risk oncogenic HPV genotypes (HR-HPV) is the established causal factor for CIN and cervical cancer development [3]. More than 200 HPV genotypes have been identified, at least 14 of which are considered high-risk, with HPV16 and HPV18 being the most frequently implicated in carcinogenesis [4]. Specifically, HPV16 has been detected in approximately 50–60% of invasive squamous cell cervical cancers, whereas HPV18 predominates in cervical adenocarcinomas and glandular lesions [5].

Historically, cytological screening programs (Pap smears) have significantly reduced cervical cancer incidence and mortality in developed countries [6]. However, the diagnostic performance of conventional cytology is limited by its relatively low sensitivity (approximately 50%) and operator dependency [7]. Consequently, incorporating HPV DNA testing has transformed screening strategies, providing superior sensitivity for detecting precancerous lesions [8]. There is also growing interest in HPV genotyping, which not only identifies the presence of the virus but also distinguishes specific genotypes with important prognostic and clinical implications.

Several studies have shown that women infected with HPV16 have a substantially higher risk of progression to CIN2 or CIN3 compared with those infected by other high-risk types, such as HPV31, 33, 52, or 58 [9]. This finding has been confirmed in population-based screening cohorts, prospective studies, and meta-analyses. For example, Schiffman et al. demonstrated in a U.S. cohort that the 10-year cumulative probability of developing CIN3 was 17% among HPV16-positive women, compared with less than 5% among women with other HR-HPV types [10]. Current risk-based management guidelines, such as those published by the American Society for Colposcopy and Cervical Pathology (ASCCP), recommend stratifying risk by considering cytological results, HPV presence and genotype, infection history, and colposcopic findings [11,12].

Therefore, the primary objective of this study was to evaluate the association between HPV genotypes and the presence of CIN in women with abnormal cervical cytology, using histology from directed biopsies as the diagnostic reference. In addition, the study aimed to assess concordance between cytological and histological findings and to describe the age and cytological distribution of different HPV genotypes. This information will improve understanding of viral factors associated with cervical disease progression in our population and support evidence-based, personalized prevention strategies.

## 2. Materials and Methods

### 2.1. Study Design

This retrospective, cross-sectional observational study analyzed secondary data from a cohort of women with abnormal cervical cytology who attended the Colposcopy Unit of the Gynecology Service at Carlos Alberto Seguín National Hospital, part of the EsSalud healthcare network in Arequipa, Peru, between January 2022 and December 2024. The primary objective was to evaluate the association between human papillomavirus (HPV) genotypes and the presence of cervical intraepithelial neoplasia (CIN), confirmed by histopathological examination. The study was conducted and reported in accordance with the STROBE (Strengthening the Reporting of Observational Studies in Epidemiology) guidelines for cross-sectional studies [13].

### 2.2. Population and Eligibility Criteria

**Figure 1:**
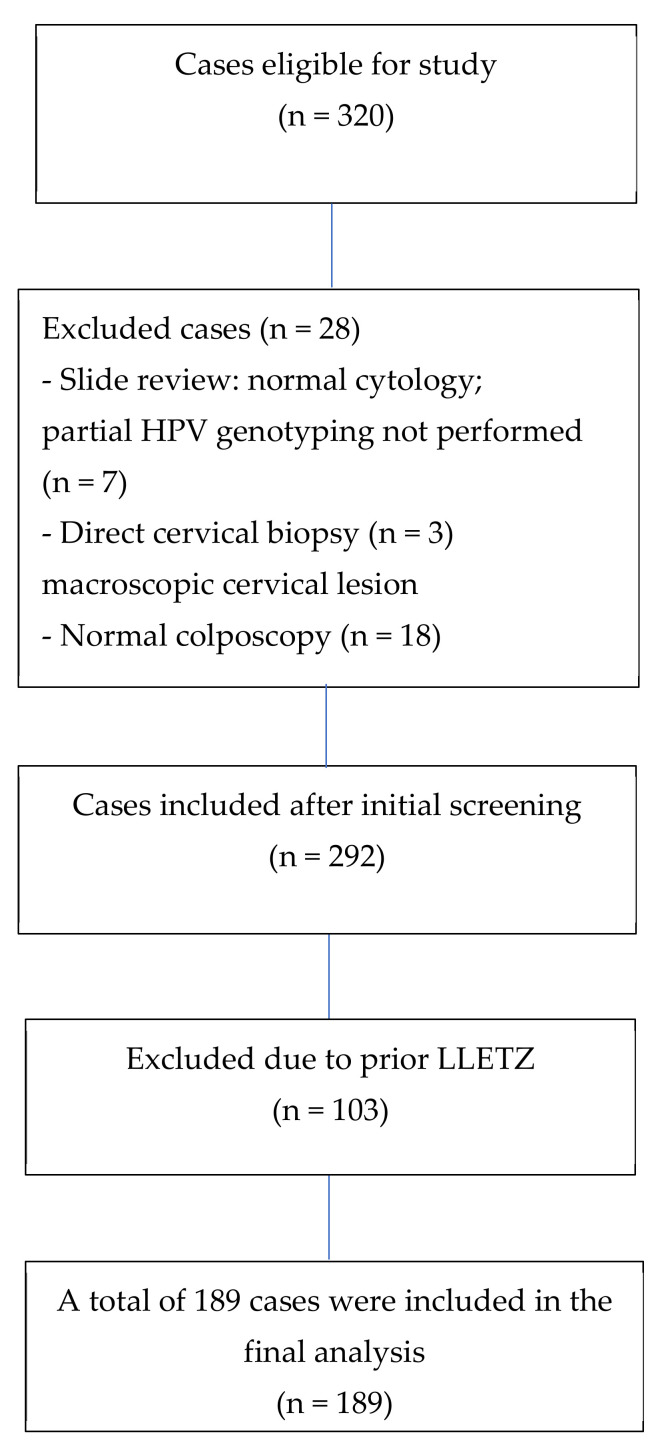
Process for the inclusion and exclusion of patients from the present study.

The study included women aged 20 to 80 years with abnormal cytological findings—including Atypical Squamous Cells of Undetermined Significance (ASC-US), Atypical Squamous Cells that cannot exclude HSIL (ASC-Hs), atypical glandular cells (AGCs), Low-Grade Squamous Intraepithelial Lesions (LSILs), or High-Grade Squamous Intraepithelial Lesions (HSILs)—who underwent partial HPV genotyping with the Cobas 4800 assays [14]. This assay detects genotypes 16 and 18, as well as 12 additional high-risk types (31, 33, 35, 39, 45, 51, 52, 56, 58, 59, 66, and 68). All participants subsequently underwent colposcopy with directed biopsy. Patients with a history of CIN treatment, cervical cancer, or known immunosuppression were excluded because of their higher and biologically distinct risk of HPV persistence and disease progression (Figure 1).

### 2.3. Study Variables

**Outcome variable:** histological diagnosis of CIN 2 or CIN 3.

**Predictor variables**:

- **HPV genotype:** categorized as negative, HPV16, HPV18, or other high-risk HPV genotypes.
- **Cytology:** classified as ASC-US, ASC-H, AGC, LSIL, or HSIL.
- **Age:** grouped into three categories: 20–29, 30–59, and ≥60 years.

### 2.4. Diagnostic Procedures

Colposcopy was performed according to a standardized protocol using a binocular colposcope. After cervical exposure, 5% acetic acid was applied, followed by Lugol’s iodine. The transformation zone and adjacent epithelium were systematically examined, and abnormal areas showing acetowhite changes or iodine negativity were biopsied under direct visualization. All procedures were conducted by accredited specialists. Targeted biopsies were obtained when colposcopic findings suggested lesions, and histopathological evaluation served as the gold standard for confirming CIN.

### 2.5. Statistical Analysis

We analyzed data with Stata 18. Descriptive statistics (frequencies and proportions) were used for categorical variables. The association between HPV genotypes and high-grade squamous intraepithelial lesions was examined using the chi-squared test. In addition, a binary logistic regression model was fitted using generalized linear models (GLMs) with a logit link function and binomial distribution, including HPV genotype and age as independent variables. Results were reported as odds ratios (ORs) with corresponding 95% confidence intervals (95% CI). A *p*-value < 0.05 was considered statistically significant.

### 2.6. Sample Size Calculation

The sample size was calculated to estimate the prevalence of high-grade squamous intraepithelial lesions (HSILs) in women with abnormal cytology. An expected prevalence of 25%, a 95% confidence level, and an absolute precision of 6% were assumed. Based on these assumptions, and using the formula for a single proportion, the required sample size was determined:

$$n_{0}=\frac{Z_{\alpha /2}^{2}p(1-p)}{d^{2}}$$

where Zα/2 = 1.96Zα/2 = 1.96, *p* = 0.25* p* = 0.25, and d = 0.06d = 0.06, and the initial sample size was 200 participants. Considering *the reference population (N = 1000), the finite population correction was applied:*

$$n=\frac{n_{0}}{1+\frac{n_{0}-1}{N}}$$

resulting in 167 participants. Finally, to compensate for an estimated 10% non-response rate, the definitive sample size was set at 186 women.

### 2.7. Ethical Considerations

This retrospective, cross-sectional observational study analyzed secondary data from a cohort of women with abnormal cervical cytology who attended the Colposcopy Unit of the Gynecology Service at Carlos Alberto Seguín National Hospital, part of the EsSalud healthcare network in Arequipa, Peru. The study protocol was reviewed and approved by the Institutional Research Ethics Committee of the hospital. All procedures adhered to the principles of the Declaration of Helsinki and complied with national and institutional regulations governing biomedical research. Because only anonymized secondary data were analyzed, the requirement for informed consent was formally waived by the ethics committee. Throughout the study, strict measures were implemented to ensure confidentiality, data security, and the responsible use of patient information.

## 3. Results

The study population consisted of 189 women. Most participants were aged 30–59 years (76.7%), indicating that midlife women comprised the majority of cases, followed by younger women aged 20–29 years (13.2%) and those ≥ 60 years (10.1%). Regarding reproductive history, multiparous women accounted for 77.8%, whereas nulliparous women represented 22.2%, indicating a predominance of women with previous childbirths (Table 1).

Regarding cytological findings, ASC-US was the most frequent abnormality (36.0%), while LSIL accounted for 28.0% and HSIL for 20.1%, highlighting a substantial proportion of both low- and high-grade lesions. ASC-H was identified in 11.6% of cases, and atypical glandular cells (AGCs) were less common (4.2%). With respect to HPV status, HPV16 was detected in 24.3% of cases and HPV18 in 2.1%, whereas other high-risk HPV genotypes predominated, being present in 73.6% of women.

**Figure 2:**
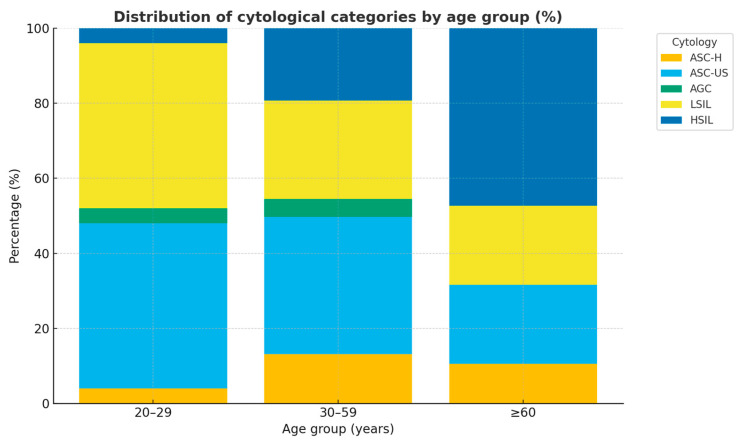
Distribution of cytological categories by age group.

Figure 2 depicts the proportional distribution of cytological categories across age groups. In women aged **20–29 years (n = 25)**, low-grade abnormalities predominated, with ASC-US and LSIL each accounting for 44.0%, while high-grade abnormalities were rare (ASC-H and HSIL, 4.0% each). Among women aged **30–59 years (n = 145)**, the most frequent abnormalities were ASC-US (36.6%) and LSIL (26.2%), followed by HSIL (19.3%) and ASC-H (13.1%), reflecting a progressive increase in high-grade lesions during midlife. In the **≥60 age group (n = 19)**, HSIL represented the largest proportion (47.4%), followed by ASC-US (21.1%) and LSIL (21.1%), with ASC-H observed less frequently (10.5%).

Overall, the findings demonstrate a clear age-related gradient. Low-grade abnormalities predominated among younger women, whereas in middle-aged women, there was a shift toward a higher prevalence of high-grade abnormalities. In women aged ≥60 years, high-grade lesions (HSILs) accounted for nearly half of all cytological diagnoses.

This distribution is consistent with the natural history of HPV infection, in which transient low-grade abnormalities are common in younger women, whereas viral persistence in older women is associated with a higher risk of progression to CIN2–3.

**Figure 3:**
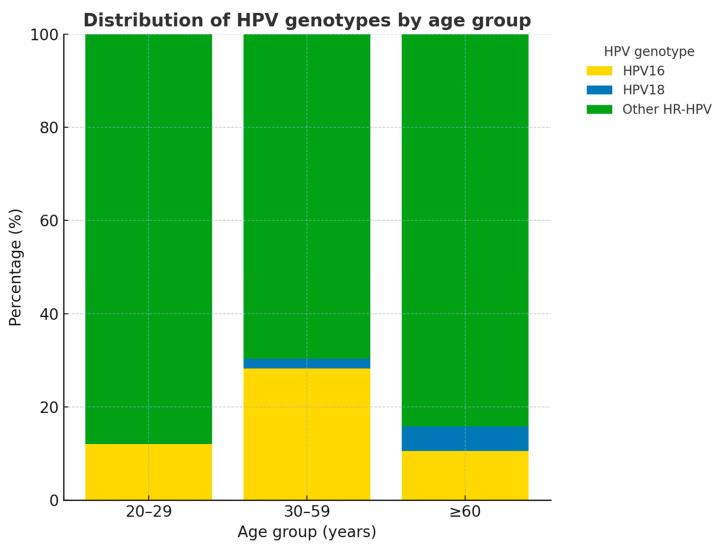
Distribution of HPV genotypes by age group.

Figure 3 shows the proportional distribution of high-risk HPV genotypes across age groups. In women aged 20–29 years (n = 25), infections were overwhelmingly dominated by other HR-HPV genotypes (88.0%), while HPV16 accounted for 12.0%, and no cases of HPV18 were observed. Among women aged 30–59 years (n = 145), the prevalence of HPV16 increased markedly (28.3%), with HPV18 detected in 2.1% of cases, and other HR-HPV genotypes representing 69.7%. This age group accounted for the largest absolute burden of HPV16 infections. In the ≥60 age group (n = 19), other HR-HPV genotypes remained predominant (84.2%), while HPV16 was less common (10.5%), and HPV18 was detected in a single case (5.3%).

In summary, the distribution reveals an age-related pattern: other hrHPV genotypes predominated in the youngest and oldest groups. HPV16 was more common in women aged 30–59 years, coinciding with the age range at highest risk for progression to high-grade cervical lesions. HPV18 was rare across all groups, with only four cases in the entire cohort (2.1%). This distribution is consistent with epidemiological evidence indicating that HPV16 is the genotype most strongly associated with high-grade cervical intraepithelial neoplasia, while other hrHPV types are more frequently detected in transient infections or low-grade abnormalities.

Table 2 summarizes the distribution of HPV genotypes across cytological categories and histological diagnoses.

In cytology, the majority of ASC-US (79.4%), LSIL (81.1%), and AGC (100.0%) cases were associated with high-risk HPV genotypes other than HPV16/18. By contrast, high-grade cytology (HSIL) showed a markedly higher prevalence of HPV16 (47.4%), equal to that of other hrHPV types (47.4%), while HPV18 was infrequent (5.3%). ASC-H also demonstrated a predominance of other hrHPV types (72.7%), although HPV16 was present in nearly one-quarter of cases (22.7%). Collectively, the distribution of HPV genotypes differed significantly among cytological categories (*p* = 0.009).

In biopsy results, the proportion of HPV16 increased progressively with lesion severity: 16.2% in negative cases, 20.0% in CIN1, 40.0% in CIN2, and 44.7% in CIN3. HPV18 remained uncommon across all groups, with only four cases detected (2.1% in total). Other hrHPV types predominated in negative and low-grade lesions (≥80%), but their frequency decreased in high-grade lesions (50.0% in CIN3). The distribution of genotypes across histological categories was statistically significant (*p* = 0.005).

Our results confirm the predominant role of HPV16 in the pathogenesis of high-grade cervical lesions (CIN2–3), while other HR-HPV types are more commonly associated with negative or low-grade abnormalities. HPV18 was rare across both cytology and biopsy, highlighting its comparatively minor contribution in this cohort.

Table 3 summarizes the distribution of cervical biopsy outcomes across age groups in 189 women with abnormal cytology. In the 20–29 age group (n = 25), the majority of biopsies were negative (72.0%), with 16.0% diagnosed as CIN1 and only a small proportion presenting with high-grade lesions (CIN2: 8.0%; CIN3: 4.0%).

Among women aged 30–59 years (n = 145), negative biopsies remained frequent (57.9%); however, the proportion of high-grade lesions was higher, with CIN2 accounting for 7.6% and CIN3 for 21.4%. CIN1 represented 13.1% of cases. In the ≥60 age group (n = 19), the prevalence of high-grade lesions was greatest: 10.5% for CIN2 and 31.6% for CIN3, while negative biopsies decreased to 47.4%, and CIN1 was identified in 10.5% of women.

On the whole, although there was a trend toward increasing severity of histological lesions with advancing age, the association between age group and biopsy outcome did not reach statistical significance (χ^2^ = 6.20, *p* = 0.401).

These findings suggest that while older women are more likely to present with high-grade lesions, particularly CIN3, the observed differences may be influenced by the relatively small number of women in the youngest and oldest age groups. Larger population-based studies are needed to confirm whether age is an independent predictor of histologically confirmed cervical intraepithelial neoplasia.

Table 4 presents the correlation between cytological categories and histological biopsy outcomes in 189 women with abnormal cytology. In **ASC-H (n = 22)**, most biopsies were negative (63.6%), while one case each was classified as CIN1 and CIN2, and 27.3% were confirmed as CIN3. Among **ASC-US (n = 68)**, the majority were negative (80.9%), with 14.7% diagnosed as CIN1 and only 4.4% as CIN2–3, underscoring the limited predictive value of ASC-US for clinically significant disease.

For **AGC (n = 8)**, half of cases were diagnosed as negative or CIN1, while one case (12.5%) corresponded to CIN3, highlighting the diagnostic variability of this cytological category. **HSIL cytology (n = 38)** demonstrated the highest concordance with histology, with 94.7% of cases confirmed as CIN2–3 and only 5.3% confirmed as negative, reaffirming its strong predictive accuracy for high-grade disease. In **LSIL cytology (n = 53)**, 69.8% of cases were negative, 18.9% were CIN1, and 11.3% were CIN2–3, suggesting a frequent overestimation of abnormal findings in low-grade cytology.

Collectively, the correlation between cytology and histology was highly significant (*p* < 0.001). These findings confirm that HSIL cytology is a reliable predictor of high-grade cervical lesions, whereas ASC-US and LSIL are associated with a high proportion of negative or low-grade outcomes, limiting their utility as indicators of clinically significant disease.

Table 5 presents the univariable and multivariable analyses of factors associated with high-grade squamous intraepithelial lesions (LIE-AG).

In the **univariable analysis**, women aged ≥30 years had a significantly higher risk of LIE-AG (OR 3.65; 95% CI 1.66–8.01; *p* = 0.001). Similarly, HPV16 infection was strongly associated with the presence of high-grade lesions (OR 3.63; 95% CI 1.76–7.50; *p* < 0.001). HPV18 showed an elevated odds ratio (OR 3.30), but this association was not statistically significant (95% CI 0.45–24.1; *p* = 0.239), likely due to the small number of cases. By contrast, infection with other high-risk HPV genotypes was inversely associated with LIE-AG (OR 0.28; 95% CI 0.13–0.56; *p* < 0.001), suggesting these genotypes were more frequently detected in negative or low-grade lesions.

In the **multivariable model**, adjusted for clinically and statistically relevant factors, both age ≥ 30 years and HPV16 positivity remained independent predictors of high-grade lesions. Women aged ≥30 years had a more than four-fold increased risk (aOR 4.50; 95% CI 1.90–10.65; *p* = 0.001), while HPV16 positivity conferred a similarly elevated risk (aOR 4.19; 95% CI 1.95–9.00; *p* < 0.001), confirming their robust association with clinically significant lesions. The model demonstrated an acceptable fit (AIC = 188.2).

Taken together, these findings underscore that age ≥ 30 years and HPV16 infection are the strongest independent predictors of high-grade cervical lesions, while other HR-HPV types appear to be associated predominantly with lower-grade abnormalities or negative findings. These results reinforce the central role of HPV16 in cervical carcinogenesis and highlight the need for genotype-specific approaches to risk stratification in clinical practice.

These results suggest that cervical screening and risk stratification should incorporate both age and comprehensive HPV genotyping, acknowledging the complexity of interactions among different viral genotypes and their specific clinical relevance in the progression to advanced precancerous cervical lesions.

## 4. Discussion

This cross-sectional study evaluated the association between HPV genotypes, cytology, and histological outcomes in 189 Latin American women with abnormal Pap smears. The majority were aged 30–59 years (76.7%) and multiparous (77.8%). Cytology revealed ASC-US (36.0%) and LSIL (28.0%) as the most frequent abnormalities, while HSIL accounted for 20.1%. HPV16 was detected in 24.3%, HPV18 in 2.1%, and other high-risk HPV types in 73.6% of cases. HSIL cytology showed >95% concordance with CIN2–3.

Analysis of baseline characteristics (Table 1) highlights the predominance of women aged 30–59 years (76.7%), reflecting the typical age distribution of patients referred for colposcopic evaluation following abnormal cytology [15]. Multiparity was frequent (77.8%). This finding is consistent with evidence linking higher parity to increased risk of cervical neoplasia, potentially through hormonal and microtraumatic pathways [16]. The cytological results revealed ASC-US (36.0%) and LSIL (28.0%) as the most common abnormalities, followed by HSIL (20.1%), underscoring the clinical challenge of differentiating transient lesions from clinically significant lesions in screening practice [17]. Regarding HPV status, other high-risk HPV genotypes predominated (73.6%), whereas HPV16 was detected in 24.3% and HPV18 in only 2.1% of cases. This distribution supports the central role of HPV16 in cervical carcinogenesis [18] while also emphasizing regional differences in genotype prevalence compared with Asian cohorts, where HPV52 and HPV58 predominate [19]. These findings underscore the importance of contextualizing screening and prevention strategies according to local epidemiology.

We identified a clear age-related gradient in the distribution of cytological abnormalities (Figure 2). Among younger women (20–29 years), low-grade lesions predominated, with ASC-US and LSIL together accounting for the majority of cases, while high-grade abnormalities were rare. In middle-aged women (30–59 years), there was a progressive increase in HSIL and ASC-H, reflecting a higher burden of clinically significant disease during this period [15]. In women aged ≥60 years, HSIL accounted for nearly half of all abnormalities, underscoring the role of viral persistence and immunosenescence in the development of advanced lesions [20,21]. This distribution aligns with the natural history of HPV infection, in which transient low-grade abnormalities are common in young women, whereas persistent infection in older women is more likely to progress to CIN2–3 [18,19].

Our study in Arequipa (Figure 3) demonstrated that HPV16 was the predominant genotype among women with abnormal cytology, detected in 24.3% of cases and strongly associated with CIN3 (20.1%). This finding is consistent with Stevens et al. in Australia, who reported HPV16 as the most prevalent genotype (35.1%) and observed its frequency increasing with lesion severity, although their cohort also showed a higher total burden of CIN3+ (36.6% vs. 20.1% in our study) [22]. In contrast, the COMPACT study by Hanley et al. in Japan focused on cytology-negative women and found HPV16/18 in only 1.3% of participants; nevertheless, HPV16/18 positivity conferred a significantly higher 12-month risk of CIN2+ compared with other high-risk HPV types [23]. In China, Song et al. reported strikingly different patterns, with non-16/18 hrHPV, particularly HPV52 and HPV58, accounting for the majority of abnormal cytology (75.8%) and contributing substantially to CIN2–3, though HPV16 remained the main driver of CIN3+ [24]. Similarly, in Sichuan, China, Mo et al. confirmed the predominance of HPV52/58/53 in their population, with HPV16 most strongly linked to high-grade lesions and invasive cancer [25]. Taken together, these comparisons highlight both the universal oncogenic dominance of HPV16 and the substantial regional variability in the contribution of other hrHPV genotypes. They underscore the importance of tailoring cervical cancer prevention strategies to local epidemiology, ensuring that vaccination programs and screening algorithms adequately reflect genotype-specific risks.

Table 2 illustrates an age-related trend in the severity of histological outcomes, with the prevalence of CIN3 increasing from 4.0% in women aged 20–29 years to 21.4% in those aged 30–59 years, and reaching 31.6% among women ≥ 60 years. Although the association did not reach statistical significance in our cohort, this pattern reflects the natural history of HPV infection, in which transient low-grade lesions predominate in younger women, whereas persistent infections are more likely to progress to high-grade disease in older ages [21,26]. Similar age-dependent gradients in CIN2–3 risk have been reported in longitudinal studies from Costa Rica and the United States, emphasizing the role of viral persistence and immune senescence in older women [20]. Our findings also align with global epidemiological data showing a second peak of cervical cancer incidence in postmenopausal women [27]. These results underscore the importance of tailored surveillance strategies in older women, particularly those without prior adequate screening, while supporting conservative management in younger women given the high rates of spontaneous regression of CIN1 and low-grade cytological abnormalities [11].

Table 3 demonstrates that HPV16 was significantly enriched in high-grade cytology and biopsy categories, with it being present in 47.4% of HSIL cases and rising from 16.2% in negative biopsies to 44.7% in CIN3. These findings are in line with global evidence confirming HPV16 as the principal oncogenic genotype, detected in 55–60% of invasive cervical cancers worldwide [28,29]. Conversely, HPV18 was rare in our cohort (2.1%) and showed limited association with squamous lesions, consistent with its recognized stronger link to glandular pathology [30,31]. Other hrHPV types predominated in ASC-US, LSIL, and negative biopsies, but their frequency declined in CIN2–3, supporting the notion that their oncogenic potential is lower compared to HPV16 [32]. Similar patterns were reported in Australia by Stevens et al., where HPV16 increased with severity, while non-16/18 genotypes were frequent in low-grade abnormalities [22]. In contrast, large Asian studies have highlighted HPV52 and HPV58 as major contributors to CIN2–3 [24,25]. Taken together, these data underscore the universal dominance of HPV16 in high-grade disease, while also emphasizing regional variability in the contribution of other hrHPV genotypes, which should be considered in prevention and vaccination strategies.

Table 4 demonstrates that HSIL cytology showed the highest concordance with histology, with more than 95% of cases confirmed as CIN2–3. This finding reinforces the reliability of HSIL as a strong predictor of clinically significant disease and supports current clinical guidelines recommending immediate colposcopy and biopsy for this category [33]. By contrast, ASC-US and LSIL cytologies were associated with high proportions of negative or CIN1 outcomes (80.9% and 69.8%, respectively), underscoring their limited predictive value for high-grade lesions and the importance of HPV testing as a triage tool in these cases [20,34]. AGC showed diagnostic variability, with only a minority progressing to CIN3, consistent with previous reports highlighting its unpredictable clinical course [35,36]. Altogether, the strong statistical association observed (χ^2^ *p* < 0.001) confirms the value of cytology in cervical cancer screening, while also emphasizing the complementary role of HPV genotyping to refine risk stratification, particularly in women with equivocal or low-grade abnormalities [37,38].

Table 5 confirms that age ≥ 30 years and HPV16 infection were the strongest independent predictors of high-grade squamous intraepithelial lesions (LIE-AG). In the multivariable model, women ≥ 30 years had a more than fourfold increased risk of CIN2–3, consistent with epidemiological evidence linking older age to persistent HPV infections and progression to advanced lesions [39,40]. HPV16 positivity also conferred a significant increase in risk (aOR 4.19), reinforcing its central oncogenic role, as reported in global studies attributing 55–60% of cervical cancers to HPV16 [41,42]. Conversely, HPV18 was rare in this cohort, precluding robust conclusions about its contribution, which aligns with previous literature describing its stronger association with adenocarcinomas rather than squamous lesions [43]. Interestingly, other high-risk HPV genotypes showed an inverse association with CIN2–3 in univariable analysis, supporting evidence that these types are more frequently linked to transient or low-grade infections with limited progression potential [18,28]. Overall, these results underscore the clinical relevance of incorporating HPV genotyping into risk stratification, particularly recognizing the disproportionate oncogenic impact of HPV16, while tailoring management strategies to age and genotype-specific risks [44].

Additionally, we observed a significant inverse association between high-grade squamous intraepithelial lesions (HSILs) and high-risk HPV genotypes other than HPV16 and HPV18. This reinforces the concept that these other genotypes, although classified as high-risk, possess relatively lower oncogenic potential compared to HPV16. Previous studies indicate that these other viral types are generally associated with transient infections or low-grade cervical intraepithelial lesions with a benign clinical course [30,31].

The high correlation between cytologies classified as HSIL and histological confirmation of CIN2–3 (>95%) in our study validates the role of this cytological category in the early identification of advanced cervical lesions. This finding supports current clinical guidelines recommending prompt referral for colposcopic evaluation and targeted biopsy in these patients [33].

This study has several limitations that should be acknowledged. First, the cross-sectional design precludes causal inference regarding the temporal relationship between HPV infection and CIN progression. Second, the relatively small sample size, particularly for HPV18, limited the statistical power to evaluate less frequent genotypes. Third, data were obtained from a single institution, potentially restricting generalizability to other populations with different epidemiological profiles, screening coverage, or vaccination rates. Moreover, information on viral load, duration of infection, coinfections, and individual immunological factors was not available, despite their recognized influence on progression risk. Future multicenter longitudinal studies are needed to validate these findings.

An additional significant limitation is the absence of data regarding individual immunological factors, long-term viral persistence, specific viral load, and coinfections with multiple HPV genotypes, elements widely recognized in the literature as determinants in cervical lesion progression and carcinogenesis [27].

From a clinical perspective, our findings strongly support the adoption of differentiated management algorithms based on the identified viral genotype. The detection of HPV16 or HPV18 warrants immediate referral for colposcopy, even in the absence of significant cytological abnormalities, consistent with the recommendations of the American Society for Colposcopy and Cervical Pathology (ASCCP) [11]. In contrast, the identification of other high-risk genotypes may justify a less invasive follow-up with repeated testing to monitor potential changes over time, thereby avoiding unnecessary interventions [42,44].

From a public health standpoint, these results support risk-stratified algorithms that prioritize HPV16 in referral pathways for women with abnormal cytology, while avoiding over-referral for non-16/18 genotypes associated with low-grade cytology. Given regional genotype distributions, vaccination policies should continue to prioritize coverage with the 9-valent vaccine while also monitoring the emergence of non-vaccine hrHPV genotypes through surveillance.

We recommend that future research should include prospective follow-up studies to quantify genotype-specific persistence and progression, with particular attention to time-to-CIN3 and competing risk analyses. Extended HPV genotyping, ideally incorporating at least types 16, 18, 31, 33, 45, 52, and 58, is needed to better characterize infections currently grouped under “other hrHPV”.

## 5. Conclusions

This study confirms that HPV16 infection and age ≥ 30 years are the strongest independent predictors of high-grade cervical intraepithelial neoplasia (CIN2–3) in Latin American women with abnormal cytology. HSIL cytology showed excellent concordance with histological CIN2–3, reinforcing its clinical value as a reliable marker for immediate colposcopic referral. In contrast, other high-risk HPV types were mainly associated with low-grade or negative outcomes, highlighting their lower oncogenic potential. These findings support genotype- and age-based risk stratification to improve cervical cancer prevention and minimize overtreatment.

## Figures and Tables

**Table 1:** Baseline characteristics of the study population (n = 189).

Variable	n (%)
**Age group (years)**	
20–29	25 (13.2)
30–59	145 (76.7)
≥60	19 (10.1)
**Parity**	
Nulliparous	42 (22.2)
Multiparous	147 (77.8)
**Cytology**	
ASC-H	22 (11.6)
ASC-US	68 (36.0)
AGC	8 (4.2)
HSIL	38 (20.1)
LSIL	53 (28.0)
**HPV status**	
HPV16	46 (24.3)
HPV18	4 (2.1)
Other HR-HPV	139 (73.6)
**Total**	189 (100)

**Table 2:** Distribution of HPV genotypes according to cytology and biopsy results.

Category	HPV16, n (%)	HPV18, n (%)	Other HR-HPV, n (%)	Total, n (%)	* *p*-Value
**Cytology**					
ASC-H	5 (22.7)	1 (4.5)	16 (72.7)	22 (100)	
ASC-US	13 (19.1)	1 (1.5)	54 (79.4)	68 (100)	
AGC	0 (0.0)	0 (0.0)	8 (100.0)	8 (100)	
HSIL	18 (47.4)	2 (5.3)	18 (47.4)	38 (100)	
LSIL	10 (18.9)	0 (0.0)	43 (81.1)	53 (100)	
**Subtotal**	46 (24.3)	4 (2.1)	139 (73.5)	189 (100)	**0.009**
**Biopsy**					
Negative	18 (16.2)	2 (1.8)	91 (82.0)	111 (100)	
CIN1	5 (20.0)	0 (0.0)	20 (80.0)	25 (100)	
CIN2	6 (40.0)	0 (0.0)	9 (60.0)	15 (100)	
CIN3	17 (44.7)	2 (5.3)	19 (50.0)	38 (100)	
**Subtotal**	46 (24.3)	4 (2.1)	139 (73.5)	189 (100)	**0.005**

* chi-square.

**Table 3:** Distribution of cervical biopsy results by age group.

Age Group (years)	Negative, n (%)	CIN1, n (%)	CIN2, n (%)	CIN3, n (%)	Total, N (%)	* *p*-Value
20–29	18 (72.0)	4 (16.0)	2 (8.0)	1 (4.0)	25 (100)	
30–59	84 (57.9)	19 (13.1)	11 (7.6)	31 (21.4)	145 (100)	
≥60	9 (47.4)	2 (10.5)	2 (10.5)	6 (31.6)	19 (100)	
**Total**	111 (58.7)	25 (13.2)	15 (7.9)	38 (20.1)	189 (100)	**0.401**

* chi-square.

**Table 4:** Concordance between cervical cytology categories and histological biopsy.

Cytology Category	Negative, n (%)	CIN1, n (%)	CIN2, n (%)	CIN3, n (%)	Total, n (%)	*p*-Value
ASC-H	14 (63.6)	1 (4.5)	1 (4.5)	6 (27.3)	22 (100)	
ASC-US	55 (80.9)	10 (14.7)	1 (1.5)	2 (2.9)	68 (100)	
AGC	3 (37.5)	4 (50.0)	0 (0.0)	1 (12.5)	8 (100)	
HSIL	2 (5.3)	0 (0.0)	9 (23.7)	27 (71.1)	38 (100)	
LSIL	37 (69.8)	10 (18.9)	4 (7.5)	2 (3.8)	53 (100)	
**Total**	111 (58.7)	25 (13.2)	15 (7.9)	38 (20.1)	189 (100)	**<0.001**

**Table 5:** Univariable and multivariable analysis of factors associated with high-grade squamous intraepithelial lesions.

Factor	Univariable OR (95% CI)	*p*-Value	Multivariable aOR (95% CI)	*p*-Value
Age ≥ 30	3.65 (1.66–8.01)	0.001	4.50 (1.90–10.65)	0.001
HPV16+	3.63 (1.76–7.50)	<0.001	4.19 (1.95–9.00)	<0.001
HPV18+	3.30 (0.45–24.1)	0.239	–	–
Other HR-HPV+	0.28 (0.13–0.56)	<0.001	–	–

Note: Variables included in the multivariable model were those with clinical or statistical relevance. AIC = 188.2; model fit was acceptable.

## Data Availability

The data presented in this study are available upon request from the corresponding author.

## List of Abbreviations

HPV	Human papillomavirus
hrHPV	High-risk human papillomavirus
CIN	Cervical intraepithelial neoplasia
CIN2–3	Cervical intraepithelial neoplasia grades 2 and 3
ASC-US	Atypical squamous cells of undetermined significance
ASC-H	Atypical squamous cells—cannot exclude HSIL
AGC	Atypical glandular cells
LSIL	Low-grade squamous intraepithelial lesion
HSIL	High-grade squamous intraepithelial lesion
aOR	Adjusted odds ratio
OR	Odds ratio
CI	Confidence interval
GLM	Generalized linear model
LLETZ	Large loop excision of the transformation zone
STROBE	Strengthening the Reporting of Observational Studies in Epidemiology
AIC	Akaike information criterion
ASCCP	American Society for Colposcopy and Cervical Pathology
